# Mapping of Bancroftian Filariasis in Cameroon: Prospects for Elimination

**DOI:** 10.1371/journal.pntd.0004001

**Published:** 2015-09-09

**Authors:** Hugues C. Nana-Djeunga, Jules B. Tchatchueng-Mbougua, Jean Bopda, Steve Mbickmen-Tchana, Nathalie Elong-Kana, Etienne Nnomzo’o, Julie Akame, Ann Tarini, Yaobi Zhang, Flobert Njiokou, Joseph Kamgno

**Affiliations:** 1 Centre for Research on Filariasis and other Tropical Diseases (CRFilMT), Yaoundé, Cameroon; 2 Parasitology and Ecology Laboratory, Department of Animal Biology and Physiology, Faculty of Science, University of Yaoundé 1, Yaoundé, Cameroon; 3 NTD Control Program, Ministry of Public Health, Yaoundé, Cameroon; 4 Helen Keller International, Yaoundé, Cameroon; 5 Helen Keller International, Regional Office for Africa, Dakar, Senegal; 6 Faculty of Medicine and Biomedical Sciences, University of Yaoundé 1, Yaoundé, Cameroon; University of Ghana, GHANA

## Abstract

**Background:**

Lymphatic filariasis (LF) is one of the most debilitating neglected tropical diseases (NTDs). It still presents as an important public health problem in many countries in the tropics. In Cameroon, where many NTDs are endemic, only scant data describing the situation regarding LF epidemiology was available. The aim of this study was to describe the current situation regarding LF infection in Cameroon, and to map this infection and accurately delineate areas where mass drug administration (MDA) was required.

**Methodology:**

The endemicity status and distribution of LF was assessed in eight of the ten Regions of Cameroon by a rapid-format card test for detection of *W*. *bancrofti* antigen (immunochromatographic test, ICT). The baseline data required to monitor the effectiveness of MDA was collected by assessing microfilariaemia in nocturnal calibrated thick blood smears in sentinel sites selected in the health districts where ICT positivity rate was ≥ 1%.

**Principal findings:**

Among the 120 health districts visited in the eight Regions during ICT survey, 106 (88.3%) were found to be endemic for LF (i.e. had ICT positivity rate ≥ 1%), with infection rate from 1.0% (95% CI: 0.2–5.5) to 20.0% (95% CI: 10–30). The overall infection rate during the night blood survey was 0.11% (95% CI: 0.08–0.16) in 11 health districts out of the 106 surveyed; the arithmetic mean for microfilaria density was 1.19 mf/ml (95% CI: 0.13–2.26) for the total population examined.

**Conclusion/significance:**

ICT card test results showed that LF was endemic in all the Regions and in about 90% of the health districts surveyed. All of these health districts qualified for MDA (i.e. ICT positivity rate ≥ 1%). Microfilariaemia data collected as part of this study provided the national program with baseline data (sentinel sites) necessary to measure the impact of MDA on the endemicity level and transmission of LF important for the 2020 deadline for global elimination.

## Author Summary

Lymphatic filariasis, commonly known as elephantiasis, is a parasitic disease caused by the filarial nematodes *Wuchereria bancrofti*, *Brugia malayi* and *Brugia timori*. It is widely distributed in the tropics where it results in a chronic and debilitating disease. Nearly 1.4 billion people in 73 countries worldwide are threatened by lymphatic filariasis, with an estimated 120 million people infected, and more than 40 million disfigured and incapacitated by the disease. Mass drug administration of appropriate chemotherapeutic agents has been successful in eliminating the infection in some endemic areas supporting the contention that global elimination of the infection has become feasible. Before targeting lymphatic filariasis for elimination, it is necessary to map its distribution in order to identify areas where treatment is required. In this present study, two surveys were carried out in each of eight Regions of Cameroon to assess the endemicity status and intensity of the infection. Lymphatic filariasis was found to be endemic in all Regions surveyed and in almost all the constituent health districts. As virtually all of these Regions and health districts were found to be eligible for MDA treatments, baseline data were also acquired that can be used by the national program for the evaluation of the success of mass drug administration on the endemicity and transmission of the disease.

## Introduction

Lymphatic filariasis (LF) is a parasitic disease caused by three species of thread-like nematode worms—*Wuchereria bancrofti*, *Brugia malayi* and *Brugia timori*—known as filariae. Of these three parasite species, *W*. *bancrofti* accounts for nearly 90 percent of LF infections worldwide. *Brugia malayi* is prevalent only in some parts of South and Southeast Asia, and *B*. *timori* is found only in Indonesia [1]. Currently, nearly 1.4 billion people in 73 countries worldwide are threatened by LF, with an estimated number of 120 million people infected, and about 40 million disfigured and incapacitated by the disease [2]. LF is a vector-borne disease; several species of *Culex*, *Anopheles*, *Aedes*, and *Mansonia* mosquitoes are known to be involved in its transmission.

LF is one of the oldest and most debilitating neglected tropical diseases (NTDs) [3]. It is one of the important public health problems that face many countries in the developing world and is considered as an indicator of poverty [4]. While the infection is usually acquired in childhood, its visible manifestations occur later in life, causing temporary or permanent disability, such as elephantiasis and hydroceles [2]. Morbidity caused by chronic LF is mostly lifelong, and the disease is considered the world’s second leading cause of permanent long-term disability after mental illness [5,6]. LF is also responsible of a huge socio-economic burden. Indeed, patients affected by elephantiasis or hydrocele are often victims of societal discrimination, and the disease impairs their educational and employment opportunities, marriage prospects, and sexual life [1,7].

Lymphatic filariasis is among the diseases targeted for elimination. The Global Programme to Eliminate Lymphatic Filariasis (GPELF) elaborated a plan to achieve the goal of eliminating LF wherever it is endemic by 2020. The elimination strategy has two components: (i) stop the spread of infection by interrupting transmission, and (ii) alleviate the suffering of affected individuals by controlling morbidity. In order to interrupt transmission, community-wide mass treatment program should be implemented to treat the entire at-risk population. The global strategy is a once-yearly single-dose of two-drug regimen utilized by communities at risk for LF, with the goal of reaching at least 65% population coverage yearly, for 5–6 years [8,9]. In Africa where onchocerciasis and LF are co-endemic, WHO recommends an annual dose of ivermectin (150 μg/kg bodyweight), in association with albendazole (400 mg) [2].

As a prerequisite of this strategy, mapping the disease appears as the first programmatic step which should be completed to assess the disease situation in the country and identify areas where MDA is required [3]. Before the current survey, very little was known about LF in Cameroon, especially its distribution. A systematic review of prevalence data revealed that Cameroon was endemic for LF but further investigations were still needed to accurately delineate the endemic areas and/or assess the infection rate [10,11]. The present study then aimed at providing a quick-and-easy estimate of endemicity status and intensity of infection in Cameroon in order to draw a countrywide LF map and help the national program to implement mass drug administration (MDA) wherever the infection endemicity level is equal or greater than 1% [12].

## Methods

### Study area and implementation unit identification

The objective of the present study was to map LF in Cameroon, and provide the national elimination program with the baseline data necessary for the evaluation of the impact of treatments on the endemicity and transmission of the disease. The health system in Cameroon has a pyramidal structure. From top to bottom, it is organized into central, intermediate (Regional) and peripheral (District) levels. Since most of the community interventions (including treatments) are implemented at the district level in Cameroon, we chose districts as the implementation units (IU) for our surveys. Actually, there are 181 health districts in Cameroon, each one divided into health areas. Health areas are made of communities or villages targeted for interventions.

Of the 10 Regions of Cameroon, eight (Adamawa, Centre, East, Littoral, North West, South, South West and West) were targeted during the current surveys. The two other Regions (North and Far North) were not included in the surveys because treatments had already been implemented and were ongoing at the beginning of the present study, based on the certainty of the presence of the disease according to historical existing data [11] and history of clinical signs. Nevertheless, baseline data were collected in sentinel sites (see below; data are available upon request) selected in four health districts (Kar-Hay, Kousseri, Pette and Yagoua) of the Far North Region, where the highest LF endemicity level, based on the historical endemicity of the disease, were in this Region [11]. In addition, LF prevalence data concerning these two Regions were retrieved from systematic review documents covering the situation of LF in Cameroon in order to draw a complete map of the disease in the country.

### Study design and sampling

The present study was carried out in two phases using two types of surveys: The first phase of the study was performed through antigenaemia survey using the BinaxNow Filariasis immunochromatographic test (ICT) (Alere Scarborough Inc, USA) to assess the LF endemicity status and distribution in Cameroon. Depending on the ICT survey results, a microfilariaemia survey using nocturnal calibrated thick blood film was carried out in communities with the highest ICT positivity rates to establish a baseline for the evaluation of the program success [13]. Although ICT can also be used for monitoring and evaluation of LF programs in sentinel sites, the classical microscopic approach is still needed for confirmation or follow-up of people positive for ICT [3,14]. In fact, antigen rates decrease more slowly than microfilariaemia rates, and can underestimate the effects of MDA, particularly after the first few rounds [15].

### Antigenaemia survey

This survey was carried out in 2009 following the WHO guidelines for rapid mapping of bancroftian filariasis in Africa [16]. From the very few published literature [10,11] and existing data on LF in Cameroon—hospital-based records of the clinical signs (hydrocele and elephantiasis)-, two villages or communities where transmission was likely to be ongoing (previous data reporting LF or clinical signs) were selected in each IU. In each of these villages or communities, having at least 50 individuals, either male or female, aged 5 years and over, were tested for daytime filarial antigenaemia using ICT—the rapid-format card test for detection of *W*. *bancrofti* antigen [17]—adhering to the manufacturer’s instructions.

At the time of this testing, a visual inspection of the clinical presentation of each enrollee was performed and the most visible clinical signs (elephantiasis and hydrocele), those most likely to be due to *W*. *bancrofti* infection, were recorded.

### Microfilariaemia survey

At the end of the antigenaemia survey, a parasitological survey was conducted in 2010 in a limited number of villages selected in health districts with ICT positivity rate equal or greater than 1%. These groups of villages or communities, termed sentinel and spot check sites, are necessary to assess the success of the control program, i.e. to measure the impact of MDA on the endemicity level and transmission of LF. One sentinel site was identified in each health district, and at least 300 individuals (either male or female) aged 5 years and over were sampled. The sampling was limited—but not exclusively—to a single community. Regarding the low population density in some health districts, a buffer zone (not larger than 1km) was established around the target community in case the number of eligible individuals was less than 300. Doing so, it was possible to examine the required number of people (300–500) in a minimum (2–3) of villages or communities close to each other and with similar epidemiological patterns. From each participant, a calibrated thick blood film was collected at night (from 10 pm to 2 am the next day) to take into account the nocturnal periodicity of *W*. *bancrofti* [18,19]. Qualified and/or trained lab technicians collected a 50μl sample of finger-prick blood from each study participant using non-heparinized capillary tubes. Blood samples were collected in absolute aseptic conditions using sterile and single use materials. Standard procedures were used for the processing and analysis of the blood samples [20]. Slides were examined independently by bright field microscopy (magnification x100), by two experienced laboratory technicians. *W*. *bancrofti* microfilariae were identified and counted and the results expressed as microfilariae per ml of blood (mf/ml) [21]. When any discrepancy was found, the preparation was re-examined by both lab technicians.

### Data analysis and map drawing

All relevant data for LF were recorded into a purpose-built Microsoft Access database and subsequently exported into *PASW Statistics* version 18 (SPSS Inc., Chicago, IL, USA) for statistical analysis. Antigenaemia and microfilariaemia endemicity levels were expressed as the percentage of infected individuals among the total number of individuals examined; the 95% confidence interval (CI) was calculated using the Wilson method not corrected for continuity [22]. The intensity of infection was computed when the microfilarial count was available as arithmetic means, and the sampling fluctuations estimated using the 95% confidence interval (CI). Chi-square, Mann-Whitney and Kruskal-Wallis tests were used to compare LF endemicity level and mean intensity of infection between Regions, health districts, sexes and age groups, respectively.

**Fig 1:**
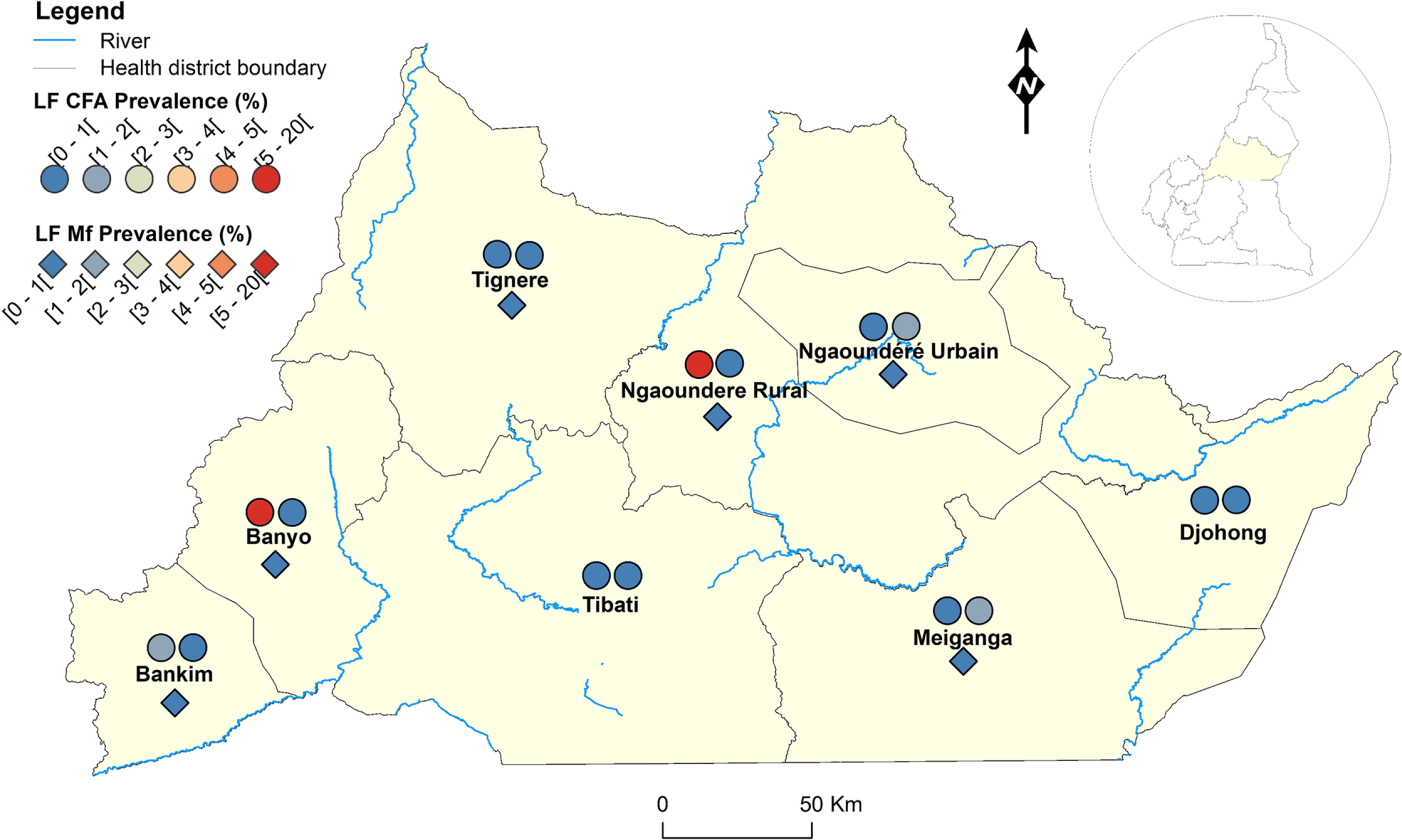
LF infection rate by circulating antigen and mf detection according to the surveyed villages in the Adamawa Region of Cameroon.

**Fig 2:**
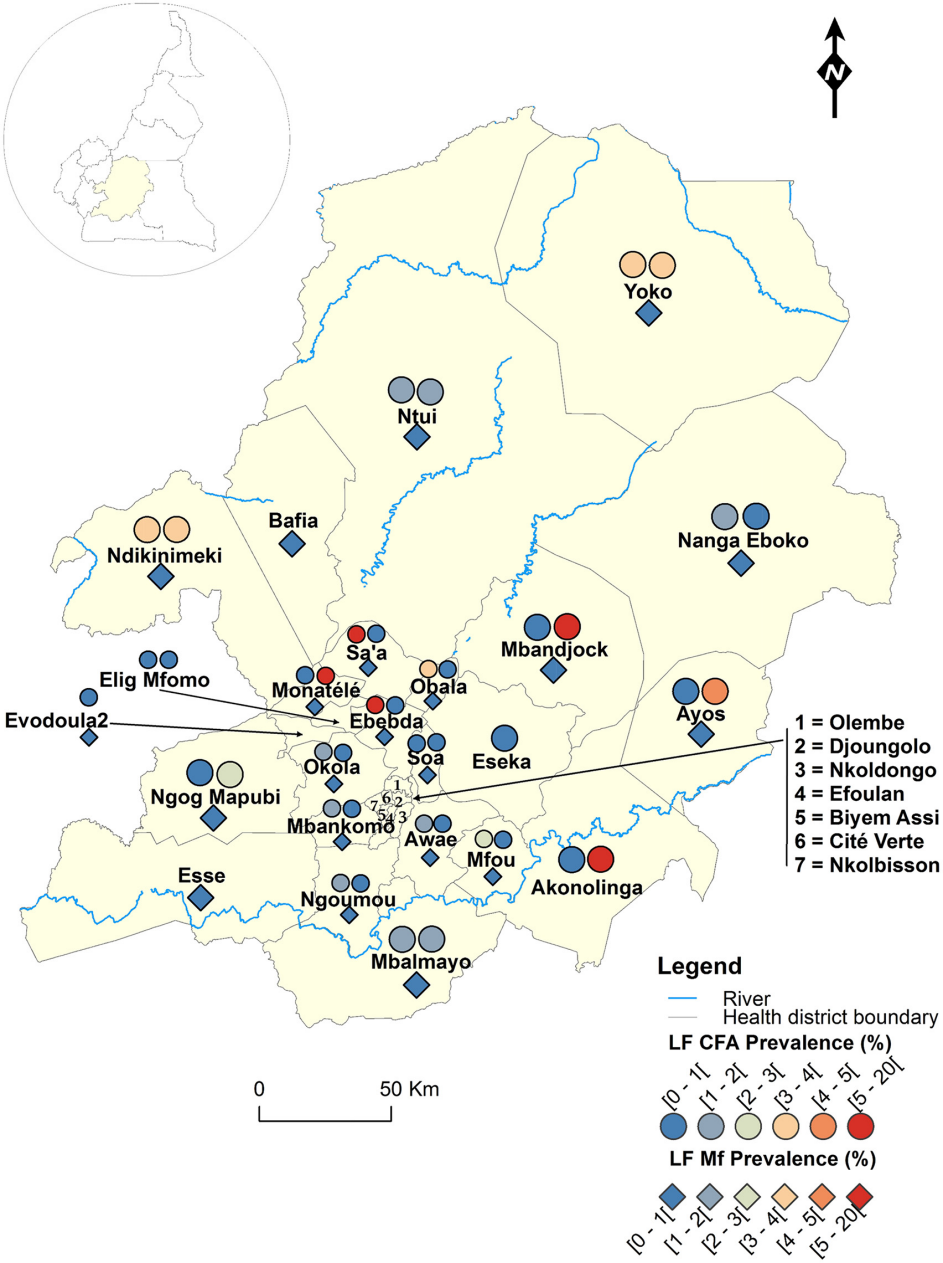
LF infection rate by circulating antigen and mf detection according to the surveyed villages in the Centre Region of Cameroon.

**Fig 3:**
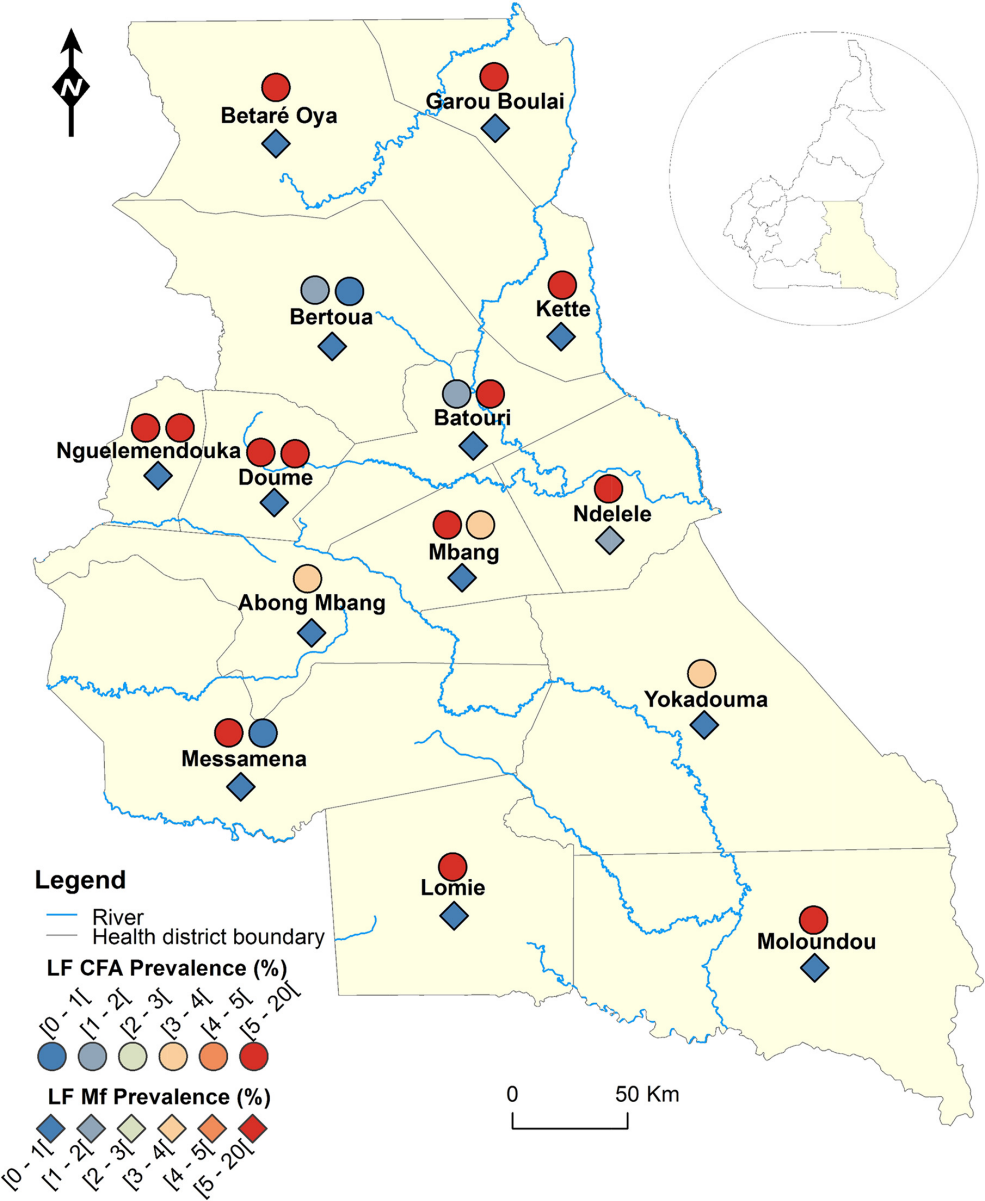
LF infection rate by circulating antigen and mf detection according to the surveyed villages in the East Region of Cameroon.

**Fig 4:**
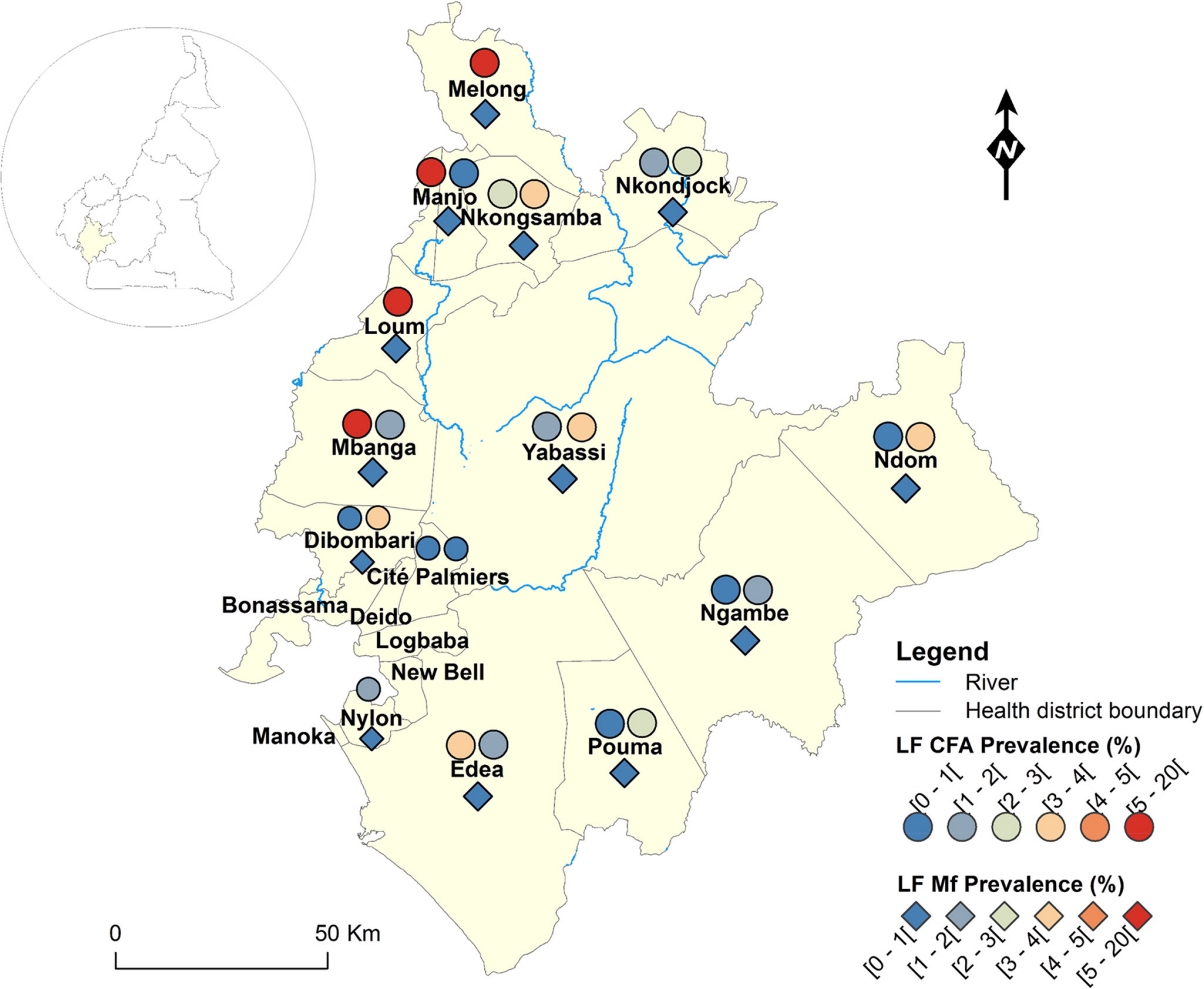
LF infection rate by circulating antigen and mf detection according to the surveyed villages in the Littoral Region of Cameroon.

**Fig 5:**
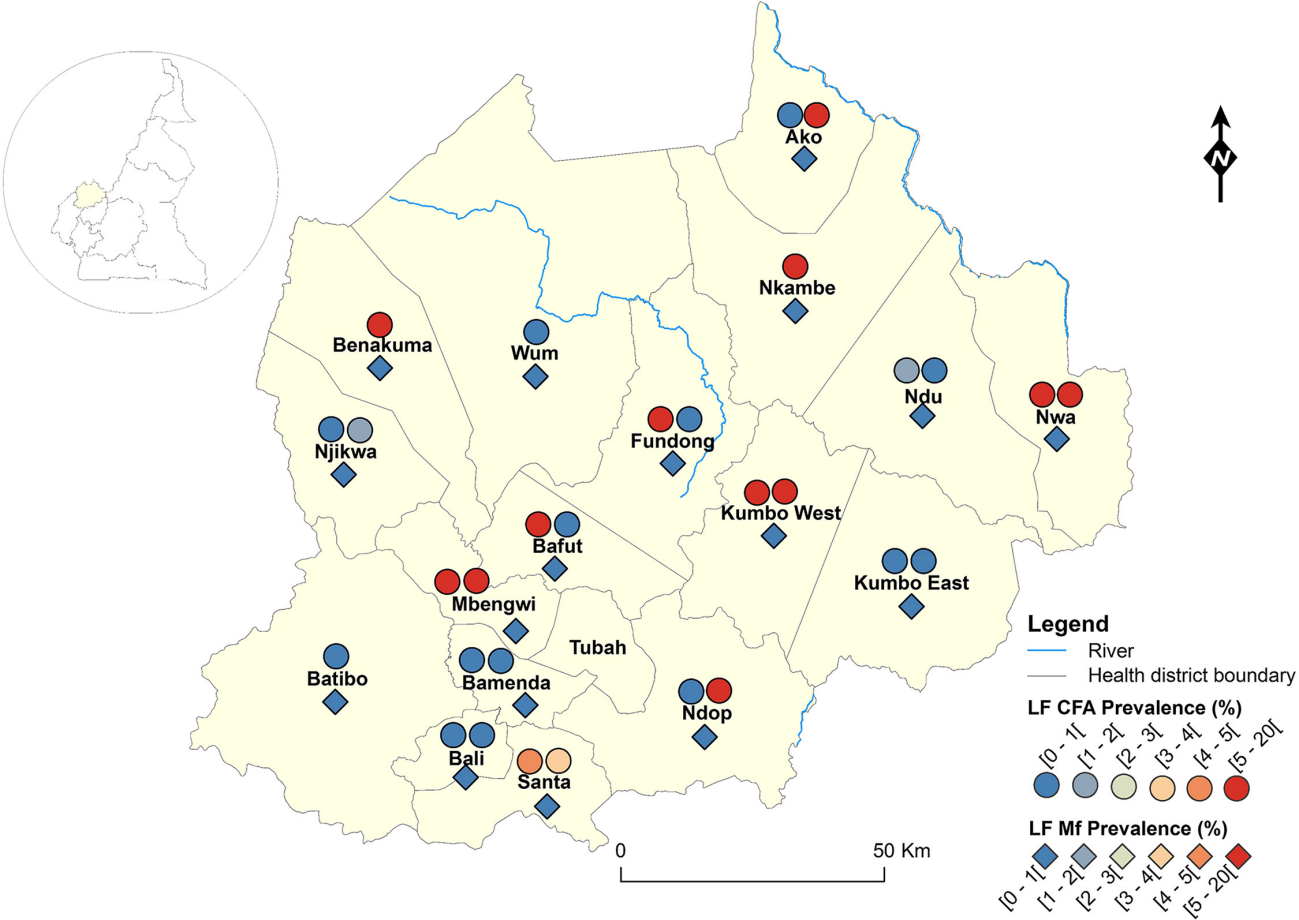
LF infection rate by circulating antigen and mf detection according to the surveyed villages in the North-West Region of Cameroon.

**Fig 6:**
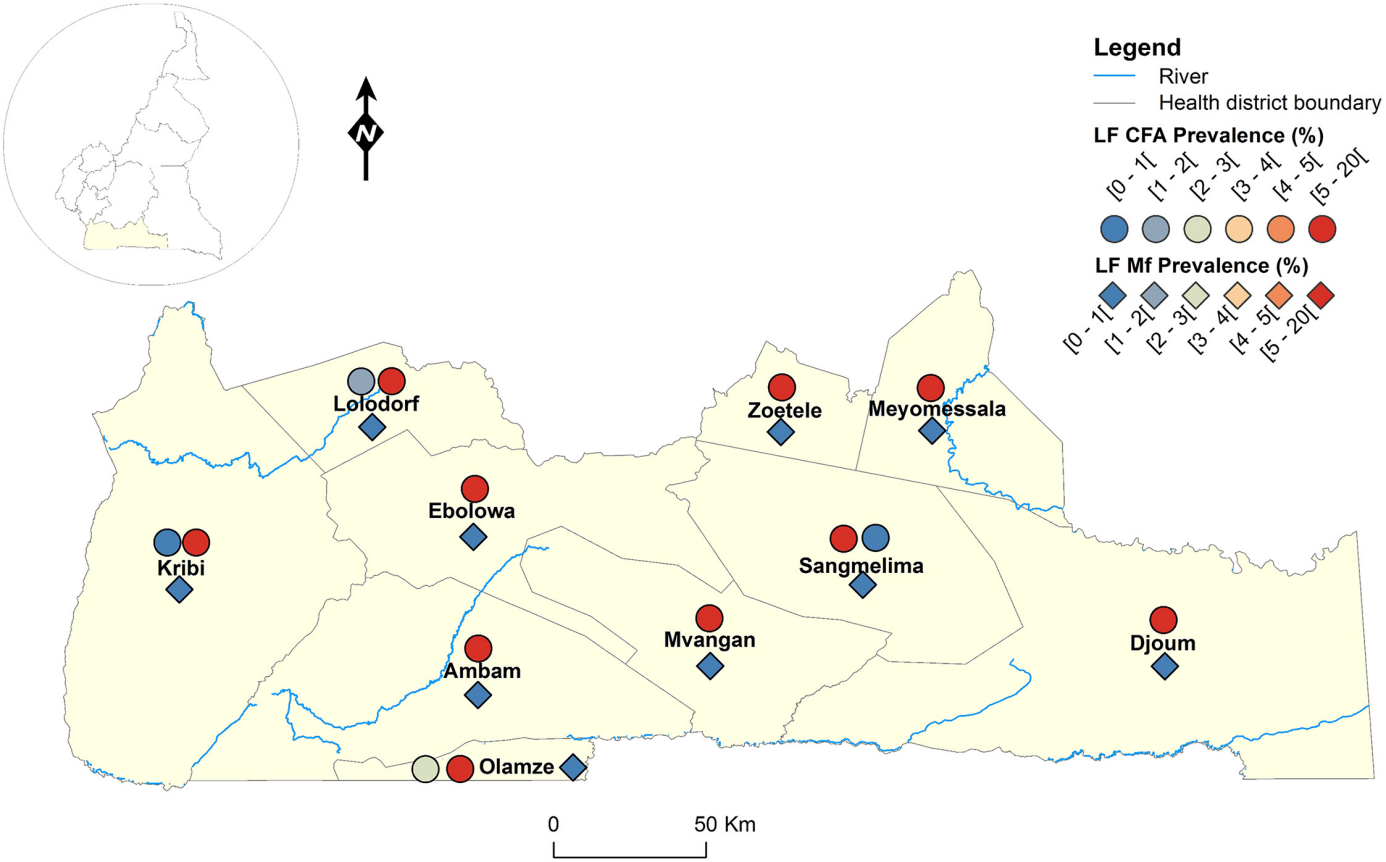
LF infection rate by circulating antigen and mf detection according to the surveyed villages in the South Region of Cameroon.

**Fig 7:**
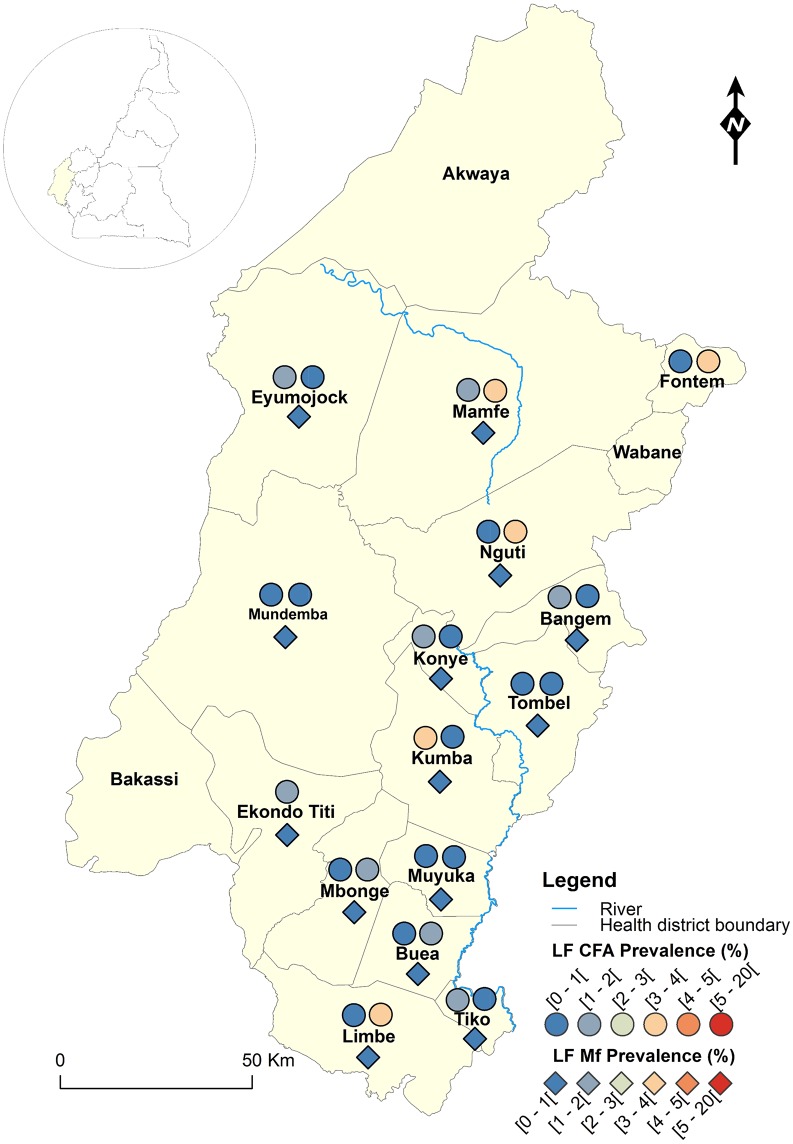
LF infection rate by circulating antigen and mf detection according to the surveyed villages in the South-West Region of Cameroon.

**Fig 8:**
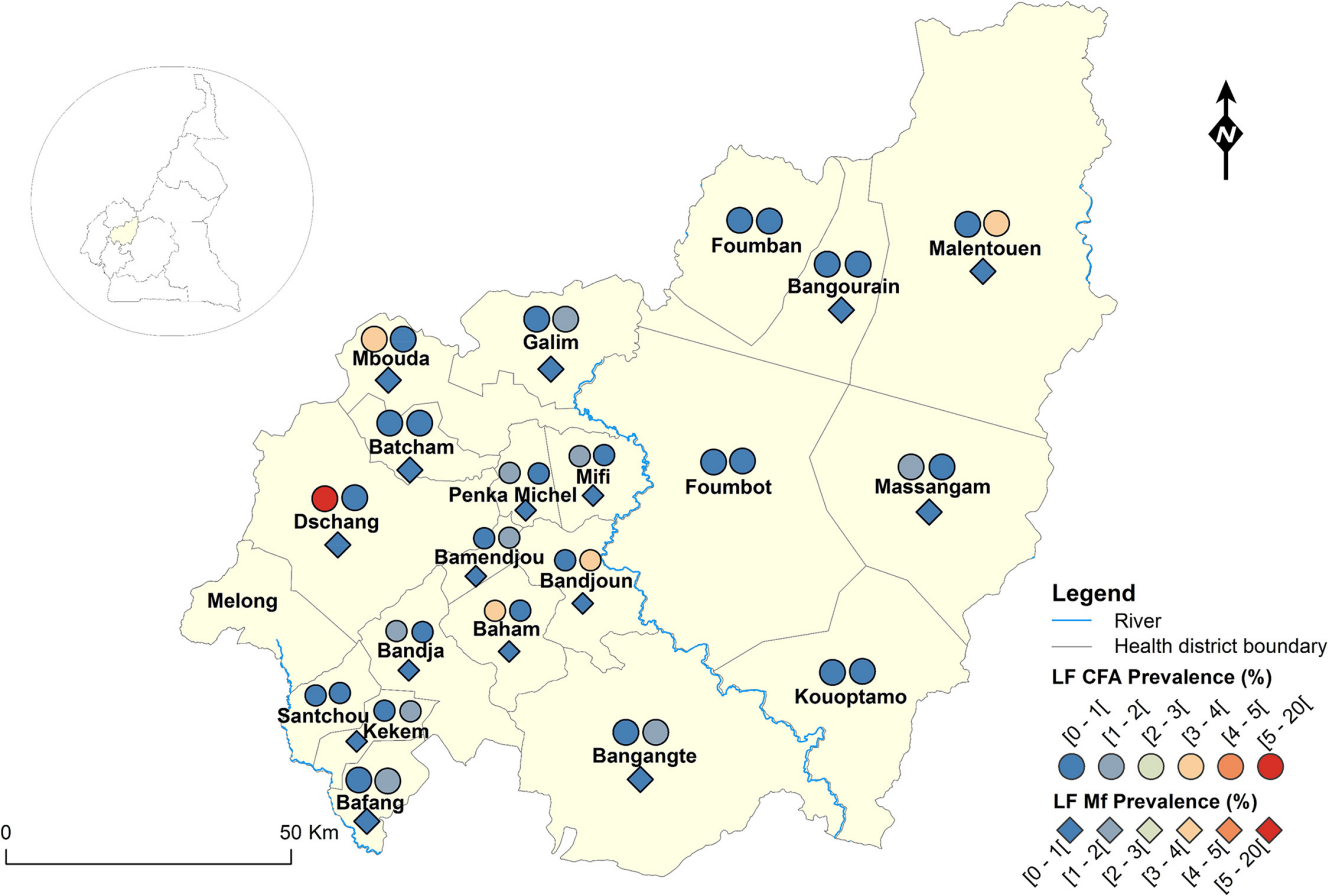
LF infection rate by circulating antigen and mf detection according to the surveyed villages in the West Region of Cameroon.

**Fig 9:**
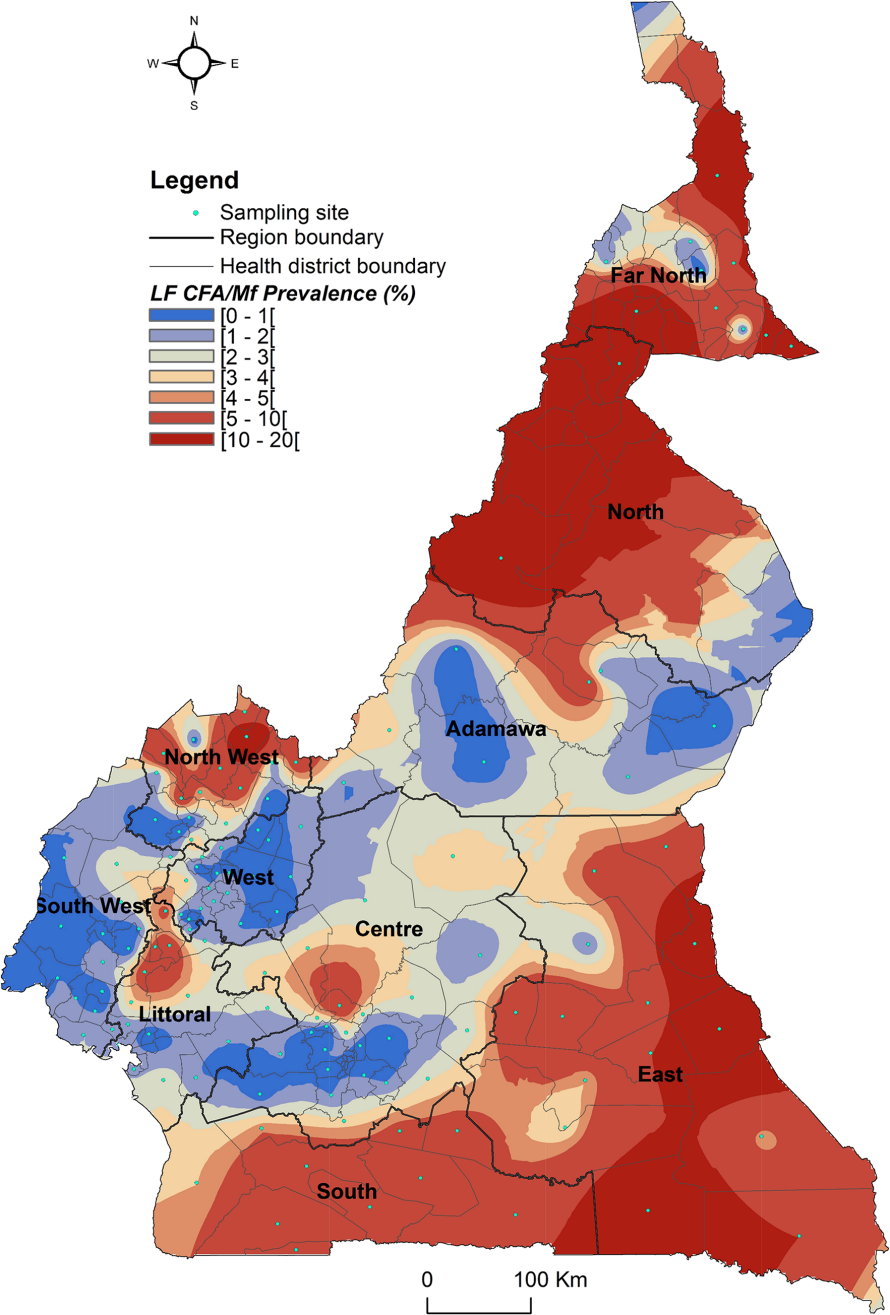
Spatially smoothed contour map predicted LF endemicity status by circulating antigen (ICT cards) and mf (night blood smears) detection in Cameroon.

The geographical coordinates of each sampled village or community were recorded using a high sensitivity global positioning system [GPS eTrex; Garmin (Europe) Ltd, Southampton, U.K.]. A thematic analysis was performed using a geographical information system (GIS) software (ArcGIS, version 10.2, ESRI Inc.) to draw the LF map in Cameroon. For each IU (health district), maps showing the LF endemicity status were drawn, for antigenaemia and microfilariaemia, and presented separately for each Region for readability requirements (Figs 1–8). An IU was considered non-endemic when the antigenaemia positivity rate was less than 1%, and endemic when the antigenaemia positivity rate was equal or greater than 1%) [3]. To provide an estimate of the situation of LF in the entire country, historical data (based on mf detection) were retrieved from published literatures [10,11] to complement the existing circulating filarial antigen (CFA) or mf data. Then, the endemicity rates (either from antigen or mf) were interpolated at the IU (health district) level using the kriging method; health districts were then delineated and a spatially smoothed contour map drawn [23,24] to provide a quick estimate of the disease endemicity in the entire country (Fig 9).

### Ethics statement

This study was conducted as part of the action plan of the national program to eliminate lymphatic filariasis in Cameroon. The surveys were approved by, and undertaken under the authority of, the Ministry of Public Health of Cameroon. After approval of the local administrative and traditional authorities, the objectives and schedules of the study were first explained to community leaders and to all eligible individuals. Because of low literacy rate in some areas and the logistics constraints related to the substantial number of individuals to be sampled, a written agreement was not requested. Verbal agreements were recommended by the Ministry of Public Health and obtained from those who agree to participate, under the discretion of community leaders. Even after the agreement of minors, the approval of their parents or legal guardians was necessary before any procedure. Each team leader was responsible to record agreements and attribute individual code to each participant for anonymous data analysis.

## Results

### Detection of circulating LF antigen with ICT card tests

A total of 10,943 individuals aged 5–100 years old (5313 males and 5630 females) were registered and examined in 2009 for the LF antigenaemia survey using ICT cards. The mean age (standard deviation, sd) of the enrollee was 37.0 (19.3) years old [males: 37.2 (19.6) and females: 36.8 (19.0)] (see S1 Dataset for detailed information).

Figs 1–8 show the LF endemicity status in each implementation unit, aggregated at the Regional (not national) level for readability requirements. Among the 120 health districts (IU) visited in the eight Regions surveyed for LF circulating antigen detection, 106 (88.3%) were found to be endemic for LF (ICT positivity rate ≥ 1%), with endemicity level increasing from 1.0% (95%CI: 0.2–5.5) to 20.0% (95%CI: 10.0–30.0). Table 1 also summarizes the crude LF endemicity status by Region, sex and age group. These results show that LF is endemic in each of the eight Regions surveyed (overall infection rate: 3.3%). The lowest infection rate was found in the West Region [1.0% (95%CI: 0.7–1.6)] and the highest one in the East Region [8.0% (95%CI: 6.5–9.9)]. Males were significantly more infected than females (*p* = 0.004) and a progressive increase in the LF endemicity level according to age was observed, adults aged 21 years and over being significantly more infected than their younger counterparts (*p*< 0.0001) (Table 1).

**Table 1:** Crude endemicity levels of LF antigenaemia and microfilariaemia, and intensity of infection by Region, sex, and age group in Cameroon.

	No persons tested by ICT cards	Percentage of antigen positives (95%CI)	No persons examined for mf	Percentage of mf positives (95%CI)	Mf density (mf/ml) (95%CI)
**Overall**	10,943	3.3 (3.0–3.7)	26,586	0.11 (0.08–0.16)	1.19 (0.13–2.26)
**By Region**					
Adamawa	724	1.7 (0.9–2.9)	1,641	0.06 (0.01–0.34)	0.07 (0.00–0.14)
Centre	2,081	2.3 (1.7–3.0)	4,431	0.23 (0.13–0.42)	3.93 (0.00–9.53)
East	998	8.0 (6.5–9.9)	3,773	0.42 (0.26–0.68)	3.75 (0.15–7.36)
Littoral	1,194	2.4 (1.7–3.5)	2,638	0.00 (0.00–0.15)	0.00 (0.00–0.00)
North West	1,679	6.7 (5.6–8.0)	4,654	0.00 (0.00–0.08)	0.00 (0.00–0.00)
South West	1,448	1.2 (0.7–1.9)	3,279	0.03 (0.01–0.17)	0.01 (0.00–0.02)
South	837	5.5 (4.2–7.3)	1,962	0.00 (0.00–0.20)	0.00 (0.00–0.00)
West	1,982	1.0 (0.7–1.6)	4,208	0.00 (0.00–0.09)	0.00 (0.00–0.00)
**By Sex**					
Male	5,313	3.8 (3.3–4.4)	12,742	0.14 (0.09–0.22)	2.03 (0.00–4.12)
Female	5,630	2.8 (2.4–3.3)	13,799	0.07 (0.04–0.13)	0.41 (0.00–1.10)
**By Age group**					
5–20	2,721	1.4 (1.0–2.0)	10,609	0.08 (0.04–0.15)	0.39 (0.00–0.87)
21–30	1,897	3.6 (2.9–4.6)	3,759	0.03 (0.01–0.16)	0.01 (0.00–0.02)
31–40	1,828	4.5 (3.6–5.5)	3,533	0.08 (0.03–0.24)	0.11 (0.00–0.27)
41–50	1,707	3.4 (2.6–4.4)	3,236	0.15 (0.06–0.35)	1.57 (0.00–4.24)
> 50	2,790	4.1 (3.4–4.9)	5,104	0.20 (0.11–0.37)	4.29 (0.00–9.48)

### Detection of *W*. *bancrofti* microfilariae with nocturnal thick blood smears

In 2010, night blood samples were collected on 26,586 individuals among which 12,742 (47.9%) were males and 13,799 were females (51.9%). The age of the enrollees varied between 5 and 110 years old, with an average of 31.2 (19.7) years old [30.1 (19.6) in males and 32.2 (19.8) in females] (see S2 Dataset for detailed information).

### Endemicity status

Figs 1–8 show endemicity status (antigenaemia and microfilaraemia) across the surveyed area, and Table 1 summarizes the crude LF positivity rate by Region, sex and age group. The LF endemicity level, assessed with mf detection in nocturnal blood smears, ranged between 0.3% and 4.0% in 11 health districts out of the 106 surveyed in the eight Regions. An average mf positivity rate of 0.11% (95% CI: 0.08–0.16) was recorded across the eight Regions included in this survey. The number of individuals harboring *W*. *bancrofti* mf was significantly higher (*p* < 0.047) in the East (0.4%) and Centre (0.2%) Regions as compared to the other Regions. The infection rate was similar between males and females (*p* = 0.120) as well as between age groups (*p* = 0.150). In the four health districts of the Far North Region not included in the mapping exercise, 404 individuals were examined for LF and CFA detected in 11 (2.7%; 95% CI: 1.5–4.8) of them.

### Intensity of infection

The variation of *W*. *bancrofti* mf densities by Region, sex and age group is given in Table 1. The overall arithmetic mean mf density was 1.2 mf/ml (95% CI: 0.1–2.3) in the total population examined. Together with the infection endemicity level, the microfilarial density was significantly higher in the Centre (3.9 mf/ml) and East (3.8 mf/ml) Regions as compared to the six other Regions surveyed (*p* < 0.0001). Although the difference was not statistically significant (*p* = 0.119), the *W*. *bancrofti* mf density tended to be higher in males (2.0 mf/ml) than in females (0.4 mf/ml). Also, the mf density was similar between age groups (*p* = 0.150), though an overall progressive increase was observed. In the Far North Region, an overall 1.7% (95% CI: 0.8–3.5) individuals harboured mf, with an intensity of infection of 7.49 mf/ml, all of the infected individuals being found in the Yagoua health district (4.9%; 95%CI: 2.4–9.7).

### Proportion of LF clinical signs

Among the 10,943 subjects examined during the ICT test survey, the visual inspection of their body revealed that 43 (0.4%) and 45 (0.4%) of them presented with the elephantiasis of the lower limbs and hydrocele, respectively. The occurrence of elephantiasis of the lower limbs was significantly higher in the Littoral (15 cases among the 43 recorded) and North-West (15 cases among the 43 recorded) Regions (*p*< 0.0001), and the proportion of individuals with hydrocele was significantly higher in the East (11 cases among the 43 recorded) and North-West (23 cases among the 43 recorded) Regions (*p*< 0.0001). The proportion of individuals with elephantiasis was even among sexes and age groups (*p*> 0.06) whereas that of hydrocele was significantly higher in individuals aged more than 50 years (*p*< 0.001).

### Spatial prediction of LF distribution in Cameroon

At the community level, the LF endemicity status from the 2009–2010 surveys or retrieved from the literature was below the 1% cut-off for 46.4% of them. Among these communities, the disease endemicity level was equal to 0.0% for 96.1%, and the highest endemicity level was 0.7% for the very few communities (3.9%) with positive cases. Above the LF endemicity threshold, the median endemicity rate was 4.0% (Minimum = 1.1%; Maximum = 23.0%), with an interquartile range equal to 5.5%. From the LF endemicity rate at the community level, the spatial distribution of LF in the entire country was predicted by interpolation (Kriging technique) (Fig 9). Although occurring at very low endemicity level, LF is endemic in almost all the country. The distribution is mostly focal, except for the East, North and South Regions, as well as some parts of the North West and Far North Regions.

## Discussion

The present study was carried out to map the distribution of LF in Cameroon and provide the national program with baseline data for the assessment of the impact of treatments on the level of endemicity and transmission of the infection.

LF endemicity status at the community level (Figs 1–8), together with the interpolation results (Fig 9), shows that the infection is widely distributed in Cameroon. All the Regions and about 90% of the health districts (MDA implementation level in Cameroon) surveyed were endemic with LF. Although the infection rate was quite low in these health districts endemic with LF, they were all qualified for MDA (CFA rate ≥ 1%). Sentinel site identification using night blood smears resulted in negative outcomes in most of the health districts surveyed. Indeed, only 11 sentinel sites were identified in the 120 implementation units included in the survey. Consequently, very few mf baseline data exist for the evaluation of post-MDA implementation. However, although ICT can underestimate the effects of MDA since antigen rates decrease more slowly in the population, particularly after the first few rounds [15,25], it can be used to evaluate the success of the LF elimination program as recommended by WHO [3].

Apart from the northern region of Cameroon (Adamawa, North and Far North), loiasis is also co-endemic in most of the southern parts of the country [24]. It was shown that ICT can be positive in individuals harboring very high Loa mf loads, even when their LF CFA is negative [26]. Then, the LF endemicity level in some communities of the southern Cameroon can be overestimated. This potential for misclassifying LF endemicity because of ICT cross-reactivity with Loa might underestimate the effects of MDA in loiasis endemic areas; therefore, it appears necessary to perform night blood smears and molecular diagnostic (using dried blood spots) from people exhibiting positive ICT as confirmation tests. However, the proportion of individuals with *W*. *bancrofti* mf in night blood smears as well as higher numbers of hydrocele individuals in these communities presented with the same trend as compare to the CFA rate. Indeed, individuals harboring mf in the nocturnal blood smears were found in four of the eight Regions surveyed, but with very low infection rate (≤ 0.06%). These results support the LF endemicity in these areas, and confirm that the antigenaemia detection with immunochromatographic tests is more sensitive than the mf detection with microcopy [23]. In addition to the poor sensitivity of microscopy especially when the intensity of infection is low, amicrofilariaemic individuals cannot be detected by blood smear examinations for mf. One of the factors which can contribute to lower the rate and intensity of infection is the treatment. Indeed no MDA was ever implemented in the eight Regions surveyed to control LF at the outset of this study. However, as onchocerciasis was shown to be widely distributed in Cameroon—all the Regions are affected with more than 6 million people infected [27,28]—ivermectin was already distributed since 1992 (up to 22 annual rounds in some areas) as part of community interventions. It was shown that mf rate and intensity for LF infections were quite low in villages regularly treated for many years with ivermectin as compared to untreated villages [29]. It is noteworthy that the Cameroon LF distribution pattern observed in this study confirms the predictions from a multivariate Bayesian generalized linear spatial model develop to map the distribution of LF across Africa [30].

Results of LF antigenaemia evaluation have shown that males were significantly more infected than females, and CFA rate was increasing with age, adults aged 21 years and over being more infected than their younger counterparts. These findings are in accordance with previous literatures and might be explained by the exposition-protection pattern of individuals [23]. In fact, because of the activities they practice, males and adults are the most exposed to mosquito bites. Also, since females and children are the most vulnerable to malaria (another mosquito transmitting disease), malaria control program has developed some control measures (bed nets distribution to pregnant women for example) which can finally protect them, as compared to males, both for malaria and lymphatic filariasis. It is important to notice however that such gender and age pattern regarding rate and intensity of LF infection assessed by microfilariaemia was not observed in our survey, as was found elsewhere [23]. This might likely be due to the very low proportion of infected individuals who, in addition, harbor very low mf densities.

LF signs observed at the occasion of ICT card tests confirm that the disease is endemic in all the Regions surveyed. Although the migration history of affected individuals was not recorded, these signs were mostly found in North-West and Littoral Region where elephantiasis of non-filarial origin (podoconiosis) was also reported [31]. This being said, the immunochromatographic card test targeting specifically adult *W*. *bancrofti* antigen circulating in the blood was positive in these Regions, and hydroceles were also noticed.

This study, together with historical data, provides the national program for elimination of LF in Cameroon with a countrywide map of the infection and highlight areas where MDA should be prioritized. These data allow the national program to implement MDA only in those health districts where loiasis is not co-endemic or where community directed treatments with ivermectin (CDTI) had already been ongoing for onchocerciasis control, although loiasis is co-endemic. Indeed, onchocerciasis maps were realized using the rapid epidemiological mapping strategy [27, 32–33], and loiasis map drawn [24] in support to onchocerciasis and LF control or elimination programs. In areas free for loiasis and where LF is endemic (ICT rate ≥ 1%) and onchocerciasis meso-endemic (20–40% nodules prevalence or 40–60 mf prevalence) or hyper-endemic (nodules prevalence > 40% or mf prevalence > 60%), annual treatments (ivermectin in combination with albendazole) are distributed to all eligible population. In areas where loiasis is endemic, CDTI can be organized in onchocerciasis meso- and hyper-endemic communities following specific recommendations [34]. However, in these loiasis endemic areas, the risk of SAE is considered to be higher as compared to the profit resulting in the treatment, regardless of LF endemicity status and when onchocerciasis is hypo-endemic (nodules prevalence < 20% or mf prevalence < 40%). In the present study, among the health districts currently untreated for LF, some (in the East and South Regions) display the highest LF and loiasis endemicity levels. For example, among the 14 health districts of the East Region, treatments are ongoing in only four of them (three being partially covered); the population at risk in the remaining untreated area being estimated at 971,000 inhabitants (about 90% of the total population of the Region). It then appears important to implement treatments in those areas in order to achieve the global target of eliminating LF by 2020 [3,35]. Indeed, from untreated health districts, the disease could be reintroduced in the neighboring health districts already freed from the disease by multiple rounds of treatments. An alternative to the currently used bi-therapy (ivermectin in combination with albendazole) can be the bi-annual treatment using albendazole alone, with an anti-vector component by the usage of long lasting insecticide nets (LLINs) [36–38]. A marked reduction in *W*. *bancrofti* infection and infectivity in humans was observed in some areas of northern Uganda [39] and in two states of Nigeria [40] where both MDA and LLINs were used to reduce transmission. This encouraged Nigeria to recently launch the Africa’s first ever nationwide co-implementation plan to defeat LF and malaria [40].

Aside for providing a countrywide LF distribution map, this study also provides the national program with the baseline parasitological data necessary to begin a national elimination program and enable the measurement of any impact of MDA on the endemicity level and transmission of LF in the defined sentinel and spot check sites.

## Supporting Information

S1 Checklist STROBE statement. (DOCX)

S1 Dataset Database for lymphatic filariasis antigenaemia survey in eight Regions of Cameroon. (XLS)

S2 Dataset Database for lymphatic filariasis microfilariaemia survey in eight Regions of Cameroon. (XLS)

## References

[1] Remme JHF, Feenstra P, Lever PR, Médici A, Morel CM, et al (2006) Tropical diseases targeted for elimination: chagas disease, lymphatic filariasis, onchocerciasis, and leprosy In: Miller M, Barrett S, Henderson DA, editors. Disease control priorities in developing countries. Second Edition ed: The World Bank and Oxford University Press pp. 322. 21250324

[2] WHO (2014) Lymphatic fialriasis. World Health Organ Fact Sheet 102.

[3] WHO (2011) Monitoring and epidemiological assessment of mass drug administration in the global programme to eliminate lymphatic filariasis: a manual for national elimination programmes. World Health Organization, Geneva WHO/HTM/NTD/PCT/2011.4.

[4] Durrheim DN, Wynd S, Liese B, Gyapong JO (2004) Editorial: Lymphatic filariasis endemicity—an indicator of poverty? Trop Med Int Health 9: 843–845. 15303987 10.1111/j.1365-3156.2004.01287.x

[5] Gyapong JO, Kumaraswami V, Biswas G, Ottesen EA (2005) Treatment strategies underpinning the global programme to eliminate lymphatic filariasis. Expert Opin Pharmacother 6: 179–200. 15757416 10.1517/14656566.6.2.179

[6] WHO (1995) Bringing the gaps. World Health Organ Tech Rep Ser, Geneva.

[7] Babu BV, Mishra S, Nayak AN (2009) Marriage, sex, and hydrocele: an ethnographic study on the effect of filarial hydrocele on conjugal life and marriageability from Orissa, India. PLoS Negl Trop Dis 3: e414 10.1371/journal.pntd.0000414 19381283 PMC2666802

[8] Plaisier AP, Stolk WA, van Oortmarssen GJ, Habbema JD (2000) Effectiveness of annual ivermectin treatment for Wuchereria bancrofti infection. Parasitol Today 16: 298–302. 10858649 10.1016/s0169-4758(00)01691-4

[9] Michael E, Malecela-Lazaro MN, Simonsen PE, Pedersen EM, Barker G, et al (2004) Mathematical modelling and the control of lymphatic filariasis. Lancet Infect Dis 4: 223–234. 15050941 10.1016/S1473-3099(04)00973-9

[10] Moyou-Somo R, Ouambe MA, Fon E, Bema J (2003) Prevalence of Bancroftian filariasis in seven villages of the Bonassama Health District in the Wouri Estuary, littoral province of Cameroon. Med Trop 63: 583–586. 15077419

[11] Boussinesq M (1999) La filariose lymphatique au Cameroun: état des connaissances. Bull liais doc OCEAC 32: 7–12.

[12] WHO (2006) Preventive chemotherapy in human helminthiasis World Health Organization, Geneva.

[13] Weil GJ, Ramzy RM (2007) Diagnostic tools for filariasis elimination programs. Trends Parasitol 23: 78–82. 17174604 10.1016/j.pt.2006.12.001

[14] Molyneux DH (2009) Filaria control and elimination: diagnostic, monitoring and surveillance needs. Trans R Soc Trop Med Hyg 103: 338–341. 10.1016/j.trstmh.2008.12.016 19181357

[15] Schuetz A, Addiss DG, Eberhard ML, Lammie PJ (2000) Evaluation of the whole blood filariasis ICT test for short-term monitoring after antifilarial treatment. Am J Trop Med Hyg 62: 502–503. 11220767 10.4269/ajtmh.2000.62.502

[16] WHO (2000) Operational guidelines for rapid mapping of bancroftian filariasis in Africa. World Health Organization, Geneva WHO/CDS/CPE/CEE/2000.9.

[17] Weil GJ, Lammie PJ, Weiss M (1997) The ICT filariasis test: a rapid-format antigen test for diagnosis of bancroftian filariasis. Parasitol Today 13: 401–404. 15275155 10.1016/s0169-4758(97)01130-7

[18] Sasa M (1976) Human filariasis. A global survey of epidemiology and control: University Park Press, Baltimore.

[19] Simonsen PE, Niemann L, Meyrowitsch DW (1997) *Wuchereria bancrofti* in Tanzania: microfilarial periodicity and effect of blood sampling time on microfilarial intensities. Trop Med Int Health 2: 153–158. 9472300 10.1046/j.1365-3156.1997.d01-237.x

[20] Organization WH (1991) Basic laboratory methods in medical parasitology Geneva: World Health Organization. 69 p.

[21] WHO (1991) Basic laboratory methods in medical parasitology World Health Organization, Geneva.

[22] Wilson EB (1927) Probable inference, the law of succession, and statistical inference. J Amer Stat Assoc 22: 209–212.

[23] Koroma JB, Bangura MM, Hodges MH, Bah MS, Zhang Y, et al (2012) Lymphatic filariasis mapping by immunochromatographic test cards and baseline microfilaria survey prior to mass drug administration in Sierra Leone. Parasit Vectors 5: 10 10.1186/1756-3305-5-10 22236419 PMC3268710

[24] Zouré HGM, Wanji S, Noma M, Amazigo UV, Diggle PJ, et al (2011) The geographic distribution of *Loa loa* in Africa: results of large-scale implementation of the Rapid Assessment Procedure for Loiasis (RAPLOA). PLoS Negl Trop Dis 5: e1210 10.1371/journal.pntd.0001210 21738809 PMC3125145

[25] Simonsen PE, Pedersen EM, Rwegoshora RT, Malecela MN, Derua YA, et al (2010) Lymphatic filariasis control in Tanzania: effect of repeated mass drug administration with ivermectin and albendazole on infection and transmission. PLoS Negl Trop Dis 4: e696 10.1371/journal.pntd.0000696 20532226 PMC2879369

[26] Bakajika D, Noigo M, Lotsima J, Masikini G, Fischer K, et al (2014) Filarial antigenemia and *Loa loa* night blood microfilaremia in an area without bancroftian filariasis in the Democratic Republic of Congo. Am J Trop Med Hyg In press. 10.4269/ajtmh.14-0358 PMC4257636 25223938

[27] Mace J, Boussinesq M, Ngoumou P, Oye J, Koeranga A, et al (1997) Country-wide rapid epidemiological mapping of onchocerciasis (REMO) in Cameroon. Annals of Tropical Medicine and Parasitology 91: 379–391. 9290845

[28] PNLO (2006) Lutte contre l'onchocercose, une affaire de tous et de chacun. In: MINSANTE, editor. Cameroun. pp. 14.

[29] Kyelem D, Sanou S, Boatin B, Medlock J, Coulibaly S, et al (2003) Impact of long-term ivermectin (Mectizan) on *Wuchereria bancrofti* and *Mansonella perstans* infections in Burkina Faso: strategic and policy implications. Ann Trop Med Parasitol 97: 827–838. 14754495 10.1179/000349803225002462

[30] Slater H, Michael E (2013) Mapping, bayesian geostatistical analysis and spatial prediction of lymphatic filariasis prevalence in Africa. PLoS One 8: e71574 10.1371/journal.pone.0071574 23951194 PMC3741112

[31] Wanji S, Tendongfor N, Esum M, Che JN, Mand S, et al (2008) Elephantiasis of non-filarial origin (podoconiosis) in the highlands of north-western Cameroon. Ann Trop Med Parasitol 102: 529–540. 10.1179/136485908X311849 18782492

[32] Ngoumou P, Walsh J, Mace J (1994) A rapid mapping technique for the prevalence and distribution of onchocerciasis: a Cameroon case study. Ann Trop Med Parasitol 88: 463–474. 7979636 10.1080/00034983.1994.11812893

[33] Noma M, Zouré H, Tekle A, Enyong P, Nwoke B, et al (2014) The geographic distribution of onchocerciasis in the 20 participating countries of the African Programme for Onchocerciasis Control: (1) priority areas for ivermectin treatment. Parasit Vectors 7: 325 10.1186/1756-3305-7-325 25053266 PMC4223657

[34] MEC, TCC (2004) Recommendations for the treatment of onchocerciasis with Mectizan in areas co-endemic for onchocerciasis and loiasis.

[35] Hoerauf A, Pfarr K, Mand S, Debrah AY, Specht S (2011) Filariasis in Africa-treatment challenges and prospects. Clin Microbiol Infect 17: 977–985. 10.1111/j.1469-0691.2011.03586.x 21722251

[36] WHO (2012) Provisional strategy for interrupting lymphatic filariasis transmission in loiasis endemic countries. World Health Organization, Geneva WHO/HTM/NTD/PCT/2012.6: 25.

[37] Kelly-Hope LA, Molyneux DH, Bockarie MJ (2013) Can malaria vector control accelerate the interruption of lymphatic filariasis transmission in Africa; capturing a window of opportunity? Parasit Vectors 6: 39 10.1186/1756-3305-6-39 23433078 PMC3599698

[38] Molyneux DH, Hopkins AD, Bradley MH, Kelly-Hope LA (2014) Multidimensional complexities of filariasis control in an era of large-scale mass drug administration programmes: a can of worms. Parasit Vectors 7: 363 10.1186/1756-3305-7-363 25128408 PMC4261528

[39] Ashton RA, Kyabayinze DJ, Opio T, Auma A, Edwards T, et al (2011) The impact of mass drug administration and long-lasting insecticidal net distribution on Wuchereria bancrofti infection in humans and mosquitoes: an observational study in northern Uganda. Parasit Vectors 4: 134 10.1186/1756-3305-4-134 21756371 PMC3158553

[40] Richards FO, Emukah E, Graves PM, Nkwocha O, Nwankwo L, et al (2013) Community-wide distribution of long-lasting insecticidal nets can halt transmission of lymphatic filariasis in southeastern Nigeria. Am J Trop Med Hyg 89: 578–587. 10.4269/ajtmh.12-0775 23939708 PMC3771303

